# An *in vitro* evaluation of the effects of desensitizing agents on microleakage of Class V cavities

**DOI:** 10.4317/jced.52755

**Published:** 2016-02-01

**Authors:** İhsan Yikilgan, Sinem Akgul, Suat Özcan, Oya Bala, Hüma Ömürlü

**Affiliations:** 1 Assistant Professor, Gazi University, Faculty of Dentistry, Department of Restorative Dentistry, Bişkek Cd. (8.Cd.) 82. Sk. No:4 06510 Emek-Ankara, Turkey; 2 Research Assistant, Gazi University, Faculty of Dentistry, Department of Restorative Dentistry, Bişkek Cd. (8.Cd.) 82. Sk. No:4 06510 Emek-Ankara, Turkey; 3 Professor, Gazi University, Faculty of Dentistry, Department of Restorative Dentistry, Bişkek Cd. (8.Cd.) 82. Sk. No:4 06510 Emek-Ankara,Turkey

## Abstract

**Background:**

The aim of this study was to evaluate the effect of a desensitizing agent on microleakage of Class V cavities.

**Material and Methods:**

72 premolar teeth were used. There were 6 groups. Class V restorations were prepared with two different restorative materials (Equia fil, GC, America and Grandio, VOCO, Germany) and two adhesive systems (Clearfil SE Bond, Kuraray, Japan and S3 Bond Plus, Kuraray, Japan) with and without desensitizing agent (Gluma Desensitizer, Heraeus Kulzer, Germany). Restorations were polished with aluminum oxide abrasive discs. Then a range of 5 - 55C thermocycling was performed 10.000 times. The microleakage of restorations was examined with dye penetration method (Basic fuchsine). Bonferroni corrections and Kruskal-Wallis test were used to determine the significance of differences in occlusal and gingival dye penetration scores between groups.

**Results:**

There was no stastistical significance between the occlusal and gingival microleakage scores within the groups were shown.

**Conclusions:**

It can be concluded that use of desensitizing agent under both high viscosity glass ionomer restorative materials and resin composites doesn’t affect the microleakage.

** Key words:**High viscosity glass ionomer cement, composite resin, desensitizing agent, microleakage.

## Introduction

Resin composites are usually preferred as restorative material on direct and indirect restorations by clinicians. Bonding to the tooth structures, the color matching, easy applicability and low cost are the benefits of resin composites. Although these benefits of resin composites some clinical failures can be observed. The main reasons of this failure are secondary caries, marginal problems, post operative sensitivity and restorative material fractures (1).

Post operative sensitivity is a short term failure that characterized with sharp pain and occurs against chemical and thermal stimulants after restoration (2). Some hypothesis that affects the post operative sensitivity have been revealed. While some researchers claim that odontoblastic extensions in the dentin tubules behaves like neuron, the others claim that neurons in the pulp reach into the dentin tubules (3,4). On the other hand, most accepted theory is hydrodynamic theory. In this theory; thermal, chemical and physical factors that affect the tooth surface cause the movement of the dentin liquid in the tubules and this results with sensitivity (5,6).

Post operative sensitivity can be occurred after many procedures such as crown preparations, amalgam fillings, tooth whitening and composite restorations (7). Post operative sensitivity seen after composite restorations can be occurred because of both removal of the smear layer and polymerization shrinkage of the materials. To prevent this case, it has recommended to use of flowable resin composites, resin modified glass ionomer cements as liner. Although, high viscosity adhesive systems, self etch adhesives and application of adhesive system as two layers are recommended techniques (8).

Post operative sensitivity is commonly seen in Class I, II and V restorations (9). Also, in Class V restorations, some clinical failures such as microleakage and loss of bonding can be seen. The aim of this study was to evaluate the effect of a desensitizing agent on microleakage of esthetic restorations and compare the sealing performance of the high viscosity glass ionomer cement with resin composites.

## Material and Methods

In this study, 72 freshly extracted non-carious premolar teeth were used. The teeth were extracted for orthodontic reasons at Gazi University Faculty of Dentistry Department of Oral and Maxillofacial Surgery. The teeth were stored in 0,9% saline solution until experimental procedures.

-Preparation of Cavities

Class V cavities were prepared on the buccal surfaces of each teeth with a diamond bur (3-mm-width, 4-mm-length and 3-mm-depth) (Diatech Diamant AG, Switzerland) under water cooling. Cavities were located under 1 mm of cemento enamel junction. Cavity depth was controlled with a millimeter-end periodontal probe.

The teeth were randomly divided into 6 groups each containing 12 teeth. The materials and contents used in this study were listed in  Table 1.

**Table 1:**
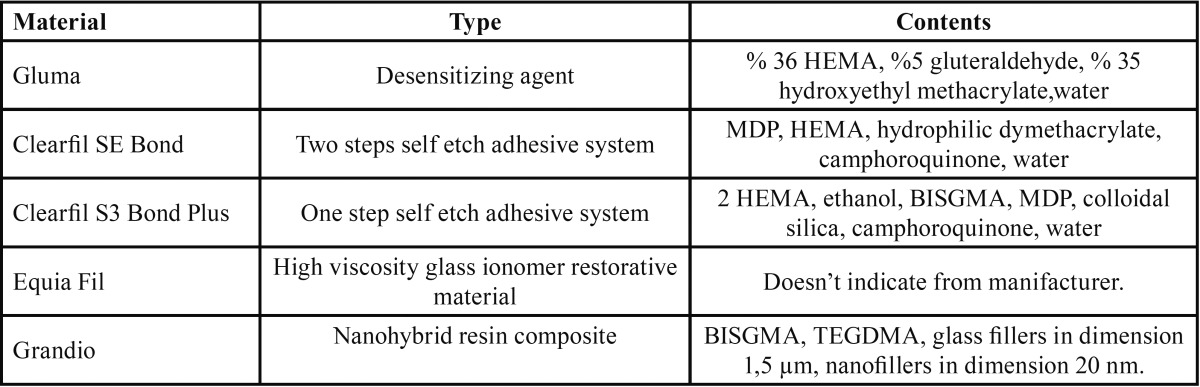
Restorative materials and contents used in this study.

Group I- After cavity preparation, high viscosity restorative material (Equia Fil, GC, Japan) was mixed in amalgamator for 10 s and applied to the cavities. Restoration surfaces were finished with a yellow band finishing bur (Meisinger, Germany) under water cooling after 2 min and 30 s working time was completed. Finally product’s own polishing agent (Equia Coat, GC, Japan) was applied on the restoration surfaces and light cured with a LED light curing unit (Elipar Freelight 2, 3M ESPE, St.Paul, MN, USA) for 20 s according to the manufacturer’s instructions.

Group II- Desensitizing agent (Gluma Desensitizer, Heraeus Kulzer, Germany) was applied to the cavities according to the maufacturer instructions (30 s air dried and rinsed under air pressure). After that, restorations were completed with same procedures as group I.

Group III- Two step self etch adhesive system (Clearfil SE Bond, Kuraray, Japan) was used. First primer was applied for 20 s and dried with mild air for 5 s. Then bond was applied to the cavity, a gentle air flow was used to make a uniform bond film and light cured for 10s with a LED light curing unit (Elipar Freelight 2, 3M ESPE, St.Paul, MN, USA). Finally nanohybrid posterior resin composite (Grandio, VOCO, Germany) was placed incrementally to the cavity and each layer was light cured for 20 s.

Group IV- After applications of desensitizing agent as in group II; the restorations were completed with same procedures as group III.

Group V- One step self etch adhesive (Clearfil S3 Bond Plus, Kuraray, Japan) was applied to the cavity for 10 s, dried with mild air for 5 s and then light cured for 10 s with a LED light curing unit (Elipar Freelight 2, 3M ESPE, St.Paul, MN, USA). Finally nanohybrid posterior resin composite (Grandio, VOCO, Germany) was placed incrementally to the cavity (Grandio, VOCO, Ger-many) and each layer was light cured for 20 s.

Group VI- After cavity preparation and desensitizing agent application, adhesive (Clearfil S3 Bond Plus, Kuraray, Japan, Grandio, VOCO, Germany) and composite resin were performed.

All restorations were polished with aluminum oxide polishing discs (Sof-Lex, 3M ESPE, America). Then all the samples were thermo cycled between 5o-55oC for 10.000 cycles.

-Microleakage evaluation

**Figure 1:**
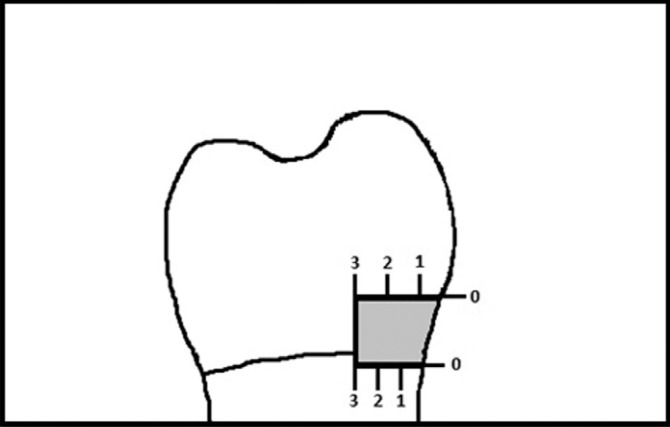
Shematic view of the microleakage scores of the samples.

The root apexes of all teeth were sealed with sticky wax and all tooth surfaces were completely covered with two layers of nail polish leaving 1 mm zone around the cavity margins. Then teeth were immersed in 0,5% basic fuchsine dye solution for 24 hours. The teeth were then rinsed under tap water and dried. They were sectioned longitudinally into two halves using a low speed saw (Mecatome T201A, Presi, Grenoble, France). All sections were evaluated for dye penetration with a stereo-microscope (Olympus SZ40, Japan) at 20x magnification. Dye penetration at the restoration/tooth interface was scored on a nonparametric scale from 0 to 3 and shown in  Table 2, Fig. 1).

**Table 2:**
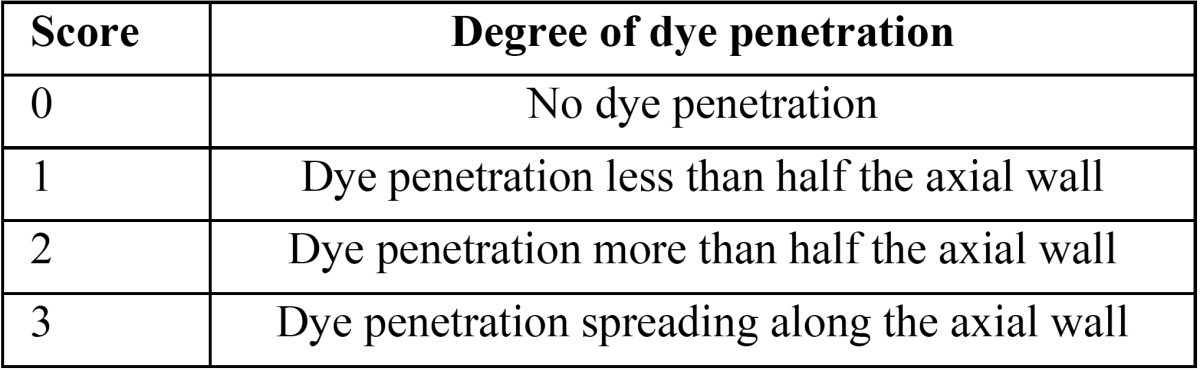
Dye penetration scores with stereomicroscope evaluation.

-Statistical Analysis

The obtained datas were recorded in SPSS (Statistical Package of Social Science) for Windows 11.5 programme. Bonferroni corrections and Kruskal-Wallis test were used to determine the significance of differences in occlusal and gingival dye penetration scores between groups. The results for *p*<0.025 were considered statitistically significant. Non- parametric multiple comparison test of Conover was used in order to determine the groups that cause to the differences when the result of Kruskal-Wallis test is significant. Mann Whitney U test was applied in order to understand whether there is a statistically significant between the occlusal and gingival dye penetration scores within the groups. According to Bonferroni correction, the results for *p*<0.0083 were con-sidered significant.

## Results

There is no statistical significance between the occlusal and gingival microleakage scores within the groups.

There is no statistical significance in terms of gingival scores within the groups (*p*=0.199).

**Figure 2:**
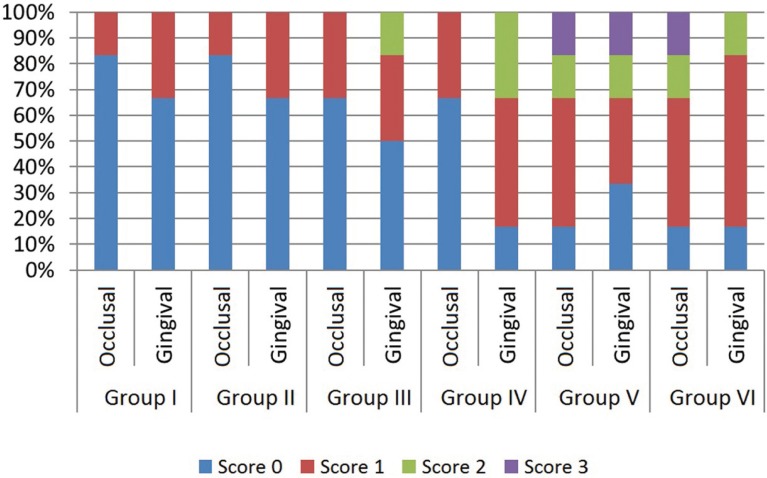
Percentage variance of the microleakage scores.

There is a significant difference at least between two groups in terms of occlusal scores (*p*= 0.018). When sub analysis was per-formed in order to examine the difference, the microleakage of Group I, II, III and IV are more significant than Group V and VI (*p*<0,025) ( Table 3). The percentage variance of microleakage scores are shown in figure 2.

**Table 3:**
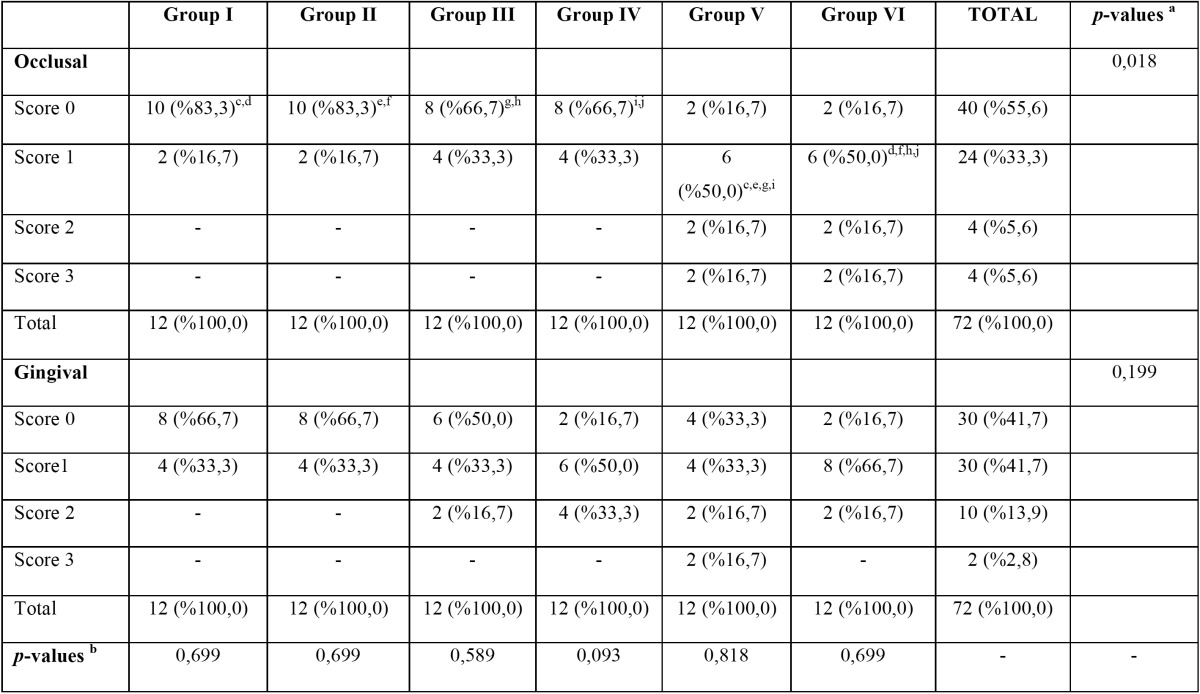
Dye penetration scores in groups, c,d. The differences between Group I and Group V, Group I and VI are statistically significant (*p*=0.003). e,f.The differences between Group II and V, Group II and VI are statistically significant (*p*=0.003). g,h,i,j. The differences between Group III and V, Group III and VI, Group IV and V, Group IV and VI are statistically significant (*P*=0.014).

## Discussion

Post-operative sensitivity is one of the main clinical problems seen after restorations. In an 8- year follow-up clinical study, Pallesen *et al.* investigated the reasons of replacement and repair of posterior restorations and showed that post operative sensitivity was the second reason after secondary caries (8). In addition, it is known that post operative sensitivity is commonly seen in Class V restorations (9). In this study, the effect of using a desensitizing agent on Class V cavities, on microleakage was evaluated.

The effects of chlorhexidine, air abrasion, beveled preparation and polymerization methods on post-operative sensitivity on Class V cavities have been researched *in-vivo* (10-15). However, the effect of desensitizing agent using post-operative sensitivity has not been researched yet *in-vivo*. The effect of desensitizing agents on bond strength of resin composites have been evaluated in previous studies *in-vitro* (16-18). There is only one study about the effect of desensitizing agents on microleakage of composite restorations *in-vitro* (19). Researchers used an oxalate containing desensitizing agent to evaluate its effect on post-operative sensitivity. Gluteraldehyde is a commonly used desensitizing agent that used to prevent post-operative sensitivity (19). In the literature, there is no study about the effect of gluteraldehyde containing desensitizing agents on the microleakage of composite resin restorations.

In literature, there are different results about the effects of desensitizing agent using on the bond strength to dentin. Some studies have indicated that using the desensitizing agent don’t affect the dentin bonding. But also in some studies, it is revealed that it increases the dentin bonding (20,21). There are few studies about the effect of desensitizing agents on the microleakage of composite resin restorations. Çelik *et al.* have evaluated the effect of two different desensitizing agents on the microleakage of servical inlays. Researchers have shown that, using HEMA and NaF containing desensitizing agents increased the microleakage on enamel surfaces (20). Shaifei *et al.* evaluated the effect of using oxalat containing desensitizing agents on the microleakage of Class V composite restorations and claimed that using desensitizing agents decrease the microleakage of some adhesives but some adhesives doesn’t affect (20). In this study, using desensitizing agent didn’t show any negative effect on adhesive systems. The effect of desensitizing agents on post-operative sensitivity and microleakage of composite resin restorations can be examined in further studies.

Within the findings of this study, there is no significant difference was shown between the groups in terms of gingival microleakage. But group V and VI were shown statistically higher microleakage values in terms of occlusal microleakage. The differences between chemical structure and application procedures of adhesives may caused these values. The main difference between one-step and two-step self-etch adhesives is NaF in the content. While one step self-etch adhesives includes NaF, two step self etch adhesives doesn’t. It has shown that NaF has negative effects on the microleakage on enamel (22). Our findings could be explained by this way. According to the manufacturer’s instructions, while the primer of two step self-etch adhesive was applied for 20 s, one step self-etch adhesive was applied for 10 s. It can be concluded that the application time can be the reason of the microleakage on the enamel surfaces.

In this study, it is observed that although high viscosity glass ionomer restorative material groups were shown higher microleakage values than one step self-etch adhesive groups, there is no significant differences between two steps self-etch adhesive groups. However, in resin composite groups, score 2 and 3 microleakage values were observed, it wasn’t observed in high viscosity glass ionomer posterior restorative material groups. Glass ionomer based materials can show hygroscopic expansion after polymerization reactions (22,23). This can affect the microleakage of high viscosity glass ionomer restorative material. There is no study which evaluates the microleakage performance of high viscosity glass ionomer restorative materials. The different aspects on microleakage performance of high viscosity glass ionomer restorative materials can be researched in further studies. These materials have positive features such as easy application to compared with other glass ionomer materials, no adhesive bonding systems need and placed bulk in cavities.

## Conclusions

According to the results of this study.

1) It can be concluded that use of desensitizing agent under both high viscosity glass ionomer restorative material and resin composites doesn’t affect the microleakage of materials.

2) High viscosity glass ionomer restorative material shows similar clinical properties resin composites in class V restorationsin terms of microleakage

3) High viscosity glass ionomer restorative material can be a suitable alternative to resin composites with some features as easy manipulation that compared with other restorative materials and need no adhesive systems for bonding.
